# Sulforaphane Enhances Cytotoxic Effects of Non-Thermal Plasma and Tirapazamine Combination Therapy in Pancreatic Adenocarcinoma Cells

**DOI:** 10.3390/cells15110975

**Published:** 2026-05-26

**Authors:** Ishfar Shaan, Brandon Gulledge, Maksym Poplavskyi, Michelle Eubank, Anastasiia Domukhovska, Anya Weinrieb, Dilbar Alseid, Isabelle Prentice, Samuel Rosen, Gamal Rayan, Shoshanna N. Zucker

**Affiliations:** 1 Jacobs School of Medicine and Biomedical Sciences, University at Buffalo, Buffalo, NY 14203, USA; ishfarsh@buffalo.edu (I.S.); meeubank@buffalo.edu (M.E.); 2 School of Pharmacy, D’Youville University, Buffalo, NY 14201, USA; gulleb18@dyc.edu (B.G.); alseid01@dyc.edu (D.A.); 3 Department of Biomedical Sciences, D’Youville University, Buffalo, NY 14201, USA; poplam01@dyc.edu (M.P.); domuka28@dyc.edu (A.D.); 4 Department of Biology, Ithaca College, Ithaca, NY 14850, USA; aweinrieb@ithaca.edu; 5 Department of Biology, D’Youville University, Buffalo, NY 14201, USA; prenti11@dyc.edu; 6 Department of Biology, Colorado College, Colorado Springs, CO 80903, USA; s_rosen2023@coloradocollege.edu; 7 Department of Drug Discovery and Biomedical Sciences, College of Pharmacy, Medical University of South Carolina, Charleston, SC 29425, USA; rayang@musc.edu; 8 Department of Pharmaceutical, Social and Administrative Sciences, School of Pharmacy, D’Youville University, Buffalo, NY 14201, USA

**Keywords:** sulforaphane, gap junctions, non-thermal plasma, tirapazamine, pancreatic adenocarcinoma

## Abstract

Pancreatic adenocarcinoma remains a highly lethal malignancy with limited effective treatment options, largely due to late-stage detection and rapid progression to metastatic disease. Therapeutic strategies capable of targeting both pre-metastatic and metastatic tumors are critically needed. In this study, we evaluated a combination therapy consisting of non-thermal plasma (NTP), a generator of reactive oxygen and nitrogen species, and the hypoxia-activated prodrug tirapazamine (TPZ). We further investigated whether sulforaphane (SF), a bioactive phytochemical, could further enhance therapeutic efficacy. NTP and TPZ produced strong cytotoxic effects as single agents and demonstrated additive to synergistic activity when combined, reducing viability by 87% in pre-metastatic BxPC-3 cells and achieving near-complete elimination of metastatic AsPC-1 cells. The addition of sulforaphane (10 µM) further enhanced cytotoxicity across all treatment conditions, with Bliss independence analysis indicating additive to synergistic interactions depending on cell line and treatment combination. Sulforaphane-mediated enhancement occurred without restoration of connexin 43 expression or coordinated reversal of epithelial-to-mesenchymal transition markers, and treatments did not induce N-cadherin upregulation or suggest acquisition of invasive characteristics. Together, these findings support NTP + TPZ as a potent combinatorial strategy for pancreatic adenocarcinoma and identify sulforaphane as an effective adjunct that enhances cytotoxic efficacy through mechanisms that remain to be fully elucidated.

## 1. Introduction

Pancreatic ductal adenocarcinoma (PDAC) remains one of the most lethal cancers, with a five-year survival rate of only 10% worldwide [1]. Currently the sixth leading cause of cancer-related deaths globally, PDAC incidence has been rising in the United States, particularly among young women [2]. The disease’s high mortality rate stems from its tendency to progress asymptomatically until advanced stages, making early detection difficult and resulting in late-stage presentation with poor survival outcomes [3]. While early-stage diagnosis significantly improves clinical prognosis by broadening therapeutic options, the majority of patients are diagnosed after the disease has progressed beyond curative intervention.

Standard treatments, such as gemcitabine-based chemotherapy, face significant challenges with drug resistance driven by metabolic reprogramming and TP53 mutations [4]. Immunotherapy, typically given in combination with chemotherapy, remains largely in clinical trials. Currently, pembrolizumab is one of the few FDA-approved immunotherapy drugs for PDAC, but is limited to patients with specific mutations, restricting its applicability [5]. Given these limitations and the rising incidence of PDAC, therapeutic approaches that can overcome resistance mechanisms and benefit a broader patient population are urgently needed. Recent advances in combinatorial strategies for PDAC have demonstrated that simultaneously targeting multiple vulnerabilities within the tumor microenvironment can overcome resistance mechanisms and enhance therapeutic efficacy, as illustrated by platinum prodrug nanoparticle systems that amplify immune activation in pancreatic cancer [6].

A novel experimental combination therapy utilizes NTP with TPZ (3-amino-1,2,4-benzotriazine-1,4-dioxide, also known as SR 4233 or WIN 59075), and has shown promise in several malignancies, including melanoma and glioblastoma [7,8]. NTP is a cold plasma generated from ionized helium that introduces reactive oxygen and nitrogen species (RONS) into cells, inducing DNA damage and cell apoptosis [7]. TPZ is a prodrug that is activated in hypoxic conditions enzymatically via NADPH-P450 reductase and xanthine oxidase, reducing TPZ to its one- and two-electron active metabolites [9,10]. Once converted to its active metabolites, TPZ functions as a potent topoisomerase II inhibitor, leading to the accumulation of DNA damage and eventually inducing apoptosis [11]. Our previous in vivo melanoma research studying combined NTP + TPZ therapy demonstrated that combining NTP and TPZ led to more severe localized melanoma tumor cell death and necrosis in NTP + TPZ treatments compared to control, NTP-, and TPZ-treated mice [11]. This synergistic effect observed in our melanoma treatment suggests that NTP + TPZ combination therapy may also be effective against PDAC tumors.

SF has demonstrated anti-cancer activity through multiple mechanisms. SF can modulate cellular redox balance, inhibit proliferative signaling pathways, enhance apoptotic responses, and sensitize cancer cells to oxidative stress-based therapies. Previous research has shown SF treatment increased connexin 43 (Cx43) expression in metastatic AsPC-1 PDAC cells, with associated reversal of epithelial-to-mesenchymal transition (EMT) [12]. The induction of EMT can lead to enhanced motility, invasion, and increased resistance to apoptotic therapies such as gemcitabine, making PDAC more difficult to treat [13,14]. Gap junctions formed by Cx43 have been implicated in the bystander effect, which can expand the area of cell death within a tumor through intercellular transport of RONS molecules and chemotherapeutic agents such as cisplatin [8,11,12,13]. Given SF’s multiple potential mechanisms of action and its reported effects on gap junction expression in PDAC cells, we investigated whether SF could enhance the therapeutic efficacy of NTP + TPZ combination therapy.

In addition to Cx43, we examined vimentin and E-cadherin as markers of EMT. Vimentin is a key biomarker of EMT, as it is a type III intermediate filament involved in cell migration and motility. It is normally expressed in mesenchymal cells but is upregulated during cancer metastasis [15]. In contrast, E-cadherin expression is lost during EMT. E-cadherin maintains cell adhesion and epithelial structural integrity, and this loss is linked to increased cancer metastasis [16]. Changes in these markers could indicate whether SF treatment affects the metastatic phenotype of PDAC cells.

The primary goal of this study was to observe the cytotoxic effects of NTP and TPZ on two PDAC cell lines: the pre-metastatic BxPC-3 and metastatic AsPC-1 cells. We tested whether the addition of SF at 10 µM, a concentration previously reported to upregulate Cx43 in AsPC-1 cells without inducing cytotoxicity [12], could enhance the cytotoxic effects of NTP + TPZ. Additionally, we explored potential mechanisms underlying any observed effects by examining changes in Cx43 expression and EMT markers to determine whether SF-mediated effects might involve modulation of gap junction communication or the metastatic phenotype.

## 2. Methods

### 2.1. IC50 of Tirapazamine and Growth Assay

The BxPC-3 and AsPC–1 cell lines were maintained in RPMI with 10% FBS and 1% penicillin/streptomycin. Tirapazamine (SR-4233) was purchased from Sigma-Aldrich (St. Louis, MO, USA). The cell lines were plated at a density of 2 × 10^5^/mL with the addition of 100 microliters per well in 2 identical 96-well plates and allowed to incubate for 24 h under normal tissue culture conditions (37 °C and 5% CO_2_). A serial dilution of TPZ was prepared in cell culture media. Media and TPZ doses were applied to the plates with 8 replicates per condition. The plates were incubated at 37 °C within the hypoxic chamber (Biospherix Model#E702m, Biospherix, Parish, NY, USA) with an O_2_ concentration of 0.1% for 24 h. Plates were assayed using a 1:10 concentration of Presto Blue (Thermo Fisher Scientific, Grand Island, NY, USA) within cell culture media and incubated for 30 min. A plate reader (BioTek Synergy HT, Winooski, VA, USA) was used to read the plates, with the fluorescence set to 530 excitation and 590 emission. The data was analyzed using GraphPad Prism (version 10) to generate IC_50_ curves.

### 2.2. IC50 of Sulforaphane and Growth Assay

The BxPC-3 and AsPC–1 cell lines were maintained in RPMI with 10% FBS and 1% penicillin/streptomycin. Sulforaphane was purchased from Sigma-Aldrich (St. Louis, MO, USA). The cell lines were plated at a density of 5 × 10^3^/mL with the addition of 100 microliters per well in 2 identical 96-well plates and allowed to incubate for 24 h under normal tissue culture conditions (37 °C and 5% CO_2_). A serial dilution of Sulforaphane was prepared in cell culture media. Media and Sulforaphane doses were applied to the plates with 8 replicates per condition. The plates were incubated at 37 °C within the hypoxia chamber (Biospherix Model#E702m, Biospherix, Parish, NY, USA) with an O_2_ concentration of 0.1% for 24 h. Plates were assayed using a 1:10 concentration of Presto Blue (Thermo Fisher Scientific, Grand Island, NY, USA) within cell culture media and incubated for 30 min. A plate reader (BioTek Synergy HT, Winooski, VA, USA) was used to read the plates, with the fluorescence set to 530 excitation and 590 emission. The data was analyzed using GraphPad Prism version 10 to generate IC50 curves.

### 2.3. Western Blot Analysis, NTP + TPZ

BxPC-3 and AsPC–1 cells were plated and treated without media for 30 s at five points per 6 cm dish, under NTP conditions at 20 V, 90.4 kHz NTP, and a helium flow rate of 4.29 L/min. Cells were treated with NTP, 20 µM TPZ, or NTP + TPZ (N/T), as indicated, and incubated for 48 h. Cell lysates were prepared with RIPA buffer, and 20 μg of each sample was loaded per well. The proteins were transferred to the PVDF membrane overnight at a constant current of 10 mA. The blots were treated with E-cadherin antibody, N-cadherin antibody, and vimentin antibody, and were normalized to GAPDH antibody. All primary antibody dilutions were 1:1000 in 3% BSA in TBS-T. All secondary antibody dilutions were 1:5000 in 5% dry milk in TBS-T. Blots were developed with ECL reagent and band intensities were quantified by densitometric analysis using ImageJ software (Version 1.54p, NIH, Bethesda, MD, USA). Western blot experiments were performed in two independent experiments using separate cell passages prepared and run on different occasions.

### 2.4. Cell Viability Readings with Addition of SF

BxPC-3 and AsPC–1 cell lines were cultured in 24-well plates for 24 h. After 24 h, the media was removed, washed with PBS, and fresh media were added. Cells being treated with SF received media containing 10 µM of SF, whereas the other wells received normal media. After a 24 h incubation, cells being treated with TPZ received a media change containing 33 µM of TPZ, while those being treated with NTP were subjected to 30 s of NTP applied to the center of the well. After another 24 h incubation, the media were changed again, but all wells received a 1:10 dilution of Presto Blue and were allowed to incubate for 30 min. The cells were read using a plate reader at a 530 nm excitation and 590 nm emission wavelength.

Drug interaction was assessed using the Bliss independence model, in which the predicted fractional inhibition of a drug combination is calculated as: E(A + B) = E(A) + E(B) − E(A) × E(B), where E(A) and E(B) represent the fractional inhibition of each agent as a single treatment. Synergy, additivity, and antagonism were defined as observed inhibition greater than, equal to, or less than predicted inhibition, respectively.

### 2.5. Western Blot Analysis with Addition of SF

BxPC-3 and AsPC–1 cells were plated and treated without media for 30 s at 5 points per 6 cm dish as described above, 20 µM TPZ, or NTP + TPZ (N/T). These experimental series were performed in the absence or presence of 10 µM SF. Cell lysates were prepared with RIPA buffer, and 40 µg was loaded per lane. The proteins were transferred overnight at a constant current of 10 mA. The blots were treated with Cx43, vimentin, E-cadherin, and GAPDH antibodies diluted to 1:1000 in blocking buffer (3% bovine serum albumin in TBS-T). The secondary antibody was diluted to 1:5000 in dry milk in TBS-T. The blots were developed with the ECL reagent. Band intensities were quantified by densitometric analysis using ImageJ software (Version 1.54p, NIH, Bethesda, MD, USA) and normalized to GAPDH as a loading control. Western blot experiments were performed in two fully independent experiments using separate cell passages prepared and run on different occasions.

## 3. Results

### 3.1. NTP and TPZ Demonstrate Additive to Synergistic Cytotoxicity in Pancreatic Adenocarcinoma Cells

**Figure 1:**
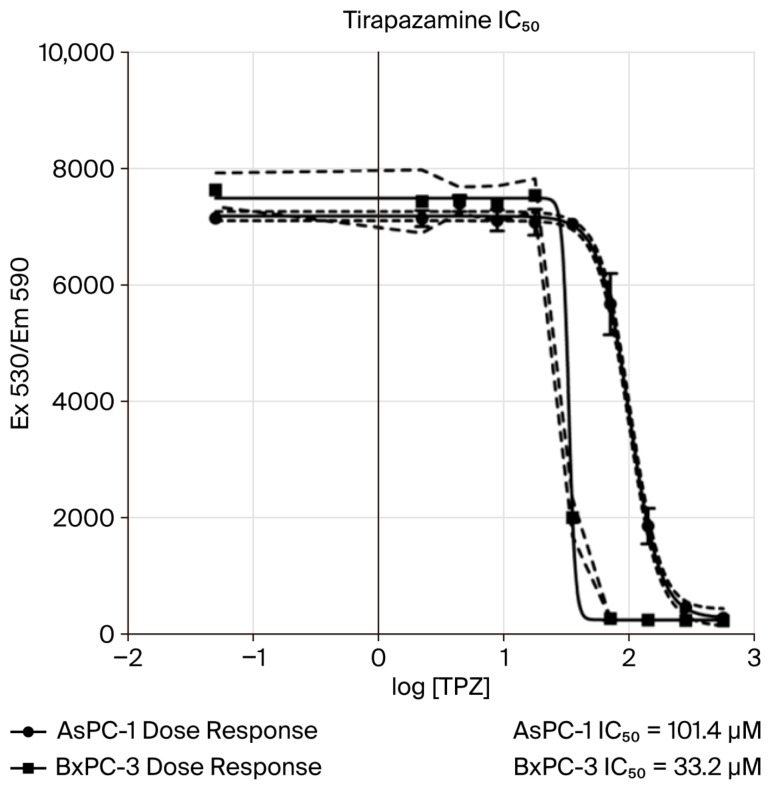
Dose–response curves for TPZ in BxPC-3 and AsPC-1 cells under hypoxic conditions (0.1% O_2_). IC_50_ values: 33.2 µM (BxPC-3) and 101.4 µM (AsPC-1).

**Figure 2:**
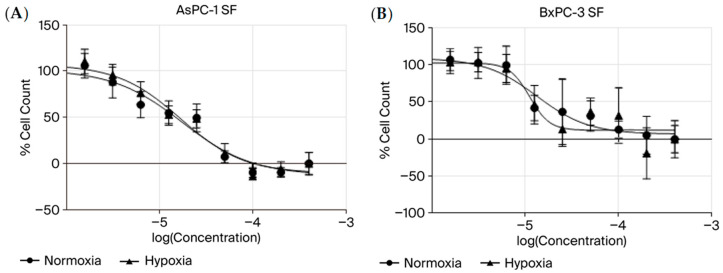
Dose–response curves for sulforaphane in AsPC-1 (**A**) and BxPC-3 (**B**) cells. (**A**) AsPC-1 IC50 values: 17.5 µM in normoxia and 16.87 µM in hypoxia. (**B**) BxPC-3 IC50 values: 11.1 µM in normoxia and 13.3 µM in hypoxia.

To evaluate the efficacy of NTP and TPZ in pancreatic adenocarcinoma with and without SF, we determined the IC_50_ values for TPZ under hypoxic conditions (0.1% O_2_) in both cell lines. Metastatic AsPC-1 cells required 101.4 µM of TPZ to achieve 50% growth inhibition, while pre-metastatic BxPC-3 cells required only 33.2 µM (Figure 1), consistent with the increased treatment resistance commonly observed in metastatic cancer cells. The IC_50_ of sulforaphane was determined to be 17.5 µM in normoxia in AsPC-1 cells (Figure 2A) and 11.1 µM in normoxia in BxPC-3 cells (Figure 2B).

**Figure 3:**
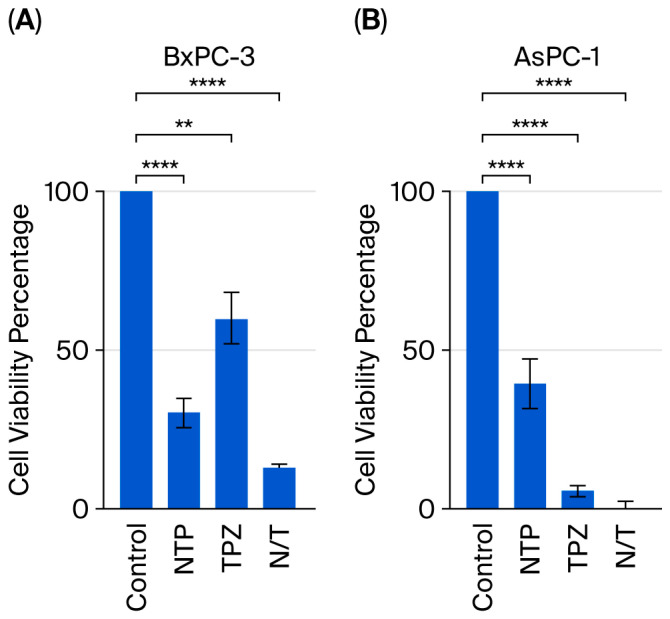
Cell viability of BxPC-3 (**A**) and AsPC-1 (**B**) cells treated with NTP, TPZ, or NTP + TPZ (N/T), expressed as a percentage relative to untreated controls (set to 100%). Bars represent mean ± SEM. Statistical significance was determined via one-way ANOVA with Dunnett’s post hoc test. ** *p* < 0.01, **** *p* < 0.0001 compared to control.

Cell viability assays demonstrated substantial cytotoxic effects of both NTP and TPZ as single agents, with reductions exceeding either single agent when combined (Figure 3, Table 1). NTP treatment alone reduced viability by 68.2% in BxPC-3 cells and 59.0% in AsPC-1 cells. TPZ treatment at IC_50_ concentrations (33 µM for BxPC-3 and 101 µM for AsPC-1) reduced viability by 40% in BxPC-3 cells and 95% in AsPC-1 cells. The combination of NTP + TPZ produced cytotoxic effects that exceeded either single agent alone, decreasing viability by 87% in BxPC-3 cells and nearly eliminating metastatic AsPC-1 cells with a 99.5% reduction in viability.

**Figure 4:**
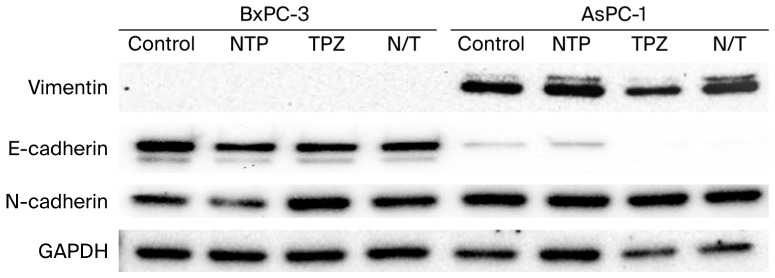
Western blot analysis of EMT markers in BxPC-3 and AsPC-1 cells treated with NTP, TPZ, or NTP + TPZ (N/T). AsPC-1 cells show faint baseline E-cadherin expression that is diminished or absent following TPZ and NTP + TPZ treatment. N-cadherin and vimentin levels remain unchanged across treatment conditions. GAPDH serves as loading control.

We examined the effects of NTP and TPZ on epithelial-to-mesenchymal transition (EMT) markers vimentin, E-cadherin, and N-cadherin by Western blot (Figure 4). Vimentin was absent in BxPC-3 cells and remained undetectable across all treatments, while AsPC-1 cells expressed vimentin at baseline with expression persisting following treatment. E-cadherin bands were clearly visible in BxPC-3 cells across all treatment conditions, while AsPC-1 cells showed faint baseline expression that was further diminished or absent following TPZ and NTP + TPZ treatment, consistent with loss of epithelial integrity commonly observed in metastatic cell lines.

N-cadherin bands were present across all treatment conditions in both cell lines with no visible changes in band intensity, and the absence of detectable N-cadherin induction combined with the lack of vimentin induction in BxPC-3 cells suggests that the typical cadherin switch associated with EMT [17] did not occur under these treatment conditions. An alternative role for E-cadherin in metastasis has been described in invasive ductal carcinoma, where E-cadherin expression can paradoxically promote metastasis by limiting reactive oxygen species-mediated apoptosis [18], which may provide context for the diminished E-cadherin band intensity observed in AsPC-1 cells following TPZ and NTP + TPZ treatment.

Collectively, these results suggest that NTP + TPZ achieves near-complete reduction in metastatic AsPC-1 cell viability (99.5%) without evidence of EMT induction, as the absence of N-cadherin upregulation and lack of vimentin induction suggest the cadherin switch did not occur under these treatment conditions. This ability to achieve potent cytotoxicity without evidence of promoting invasive characteristics in vitro represents a therapeutically favorable outcome.

### 3.2. Sulforaphane Enhances NTP + TPZ Cytotoxicity Without Coordinated EMT Reversal

#### 3.2.1. Sulforaphane Increases Cytotoxicity Across All Treatment Conditions

**Figure 5:**
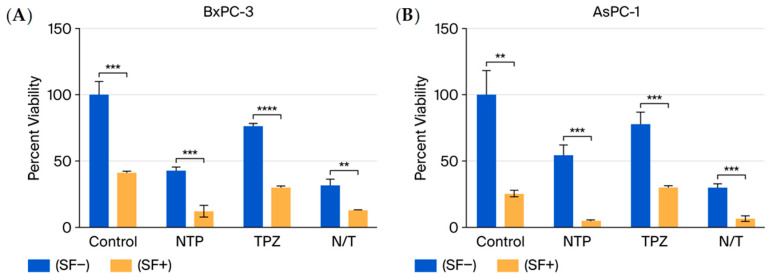
Cell viability in BxPC-3 (**A**) and AsPC-1 (**B**) cells treated with NTP, TPZ, and NTP + TPZ (N/T) in the absence or presence of 10 µM SF. In BxPC-3 cells, addition of 10 µM SF decreased control viability by ~60% (*p* = 0.000611), with ~70% decrease with TPZ (*p* = 0.000461), ~88% decrease with NTP (*p* = 0.000002), and ~87% decrease with NTP + TPZ (*p* = 0.003201). In AsPC-1 cells, addition of 10 µM SF reduced control viability by ~74% (*p* = 0.002366), with ~91% decrease with TPZ (*p* = 0.000369), ~61% decrease with NTP (*p* = 0.000796), and ~76% decrease with NTP + TPZ (*p* = 0.000568). Statistical significance was determined via one-way ANOVA with Dunnett’s post hoc test. ** *p* < 0.01, *** *p* < 0.001, **** *p* < 0.0001 compared to control.

The addition of 10 µM SF significantly enhanced cytotoxicity across all treatment conditions in both cell lines. In BxPC-3 cells, SF reduced viability from 76.7% to 30.6% with TPZ, from 43.0% to 12.5% with NTP, and from 31.6% to 13.4% with NTP + TPZ (Figure 5A, Table 2). In AsPC-1 cells, SF produced even more pronounced effects, with viability decreasing from 78.2% to 30.6% with TPZ, from 54.7% to 5.0% with NTP, and from 29.9% to 7.1% with NTP + TPZ (Figure 5B, Table 2). Bliss independence analysis revealed additive effects of SF with NTP alone (Δ + 5.2%) and TPZ alone (Δ + 0.95%) in BxPC-3 cells, but strong synergy with the NTP + TPZ combination (Δ + 27.8%). In AsPC-1 cells, SF showed moderate synergy with NTP (Δ + 9.2%), antagonism with TPZ alone (Δ − 10.3%), and additive effects with NTP + TPZ (Δ + 0.7%). Despite the antagonistic interaction between SF and TPZ in AsPC-1 cells, overall cytotoxicity remained substantial, with SF addition decreasing viability by an additional 32% in BxPC-3 cells and 40% in AsPC-1 cells on average compared to treatments without SF.

#### 3.2.2. Sulforaphane Does Not Restore Cx43 Expression or Consistently Reverse EMT Markers in Metastatic Cells

**Figure 6:**
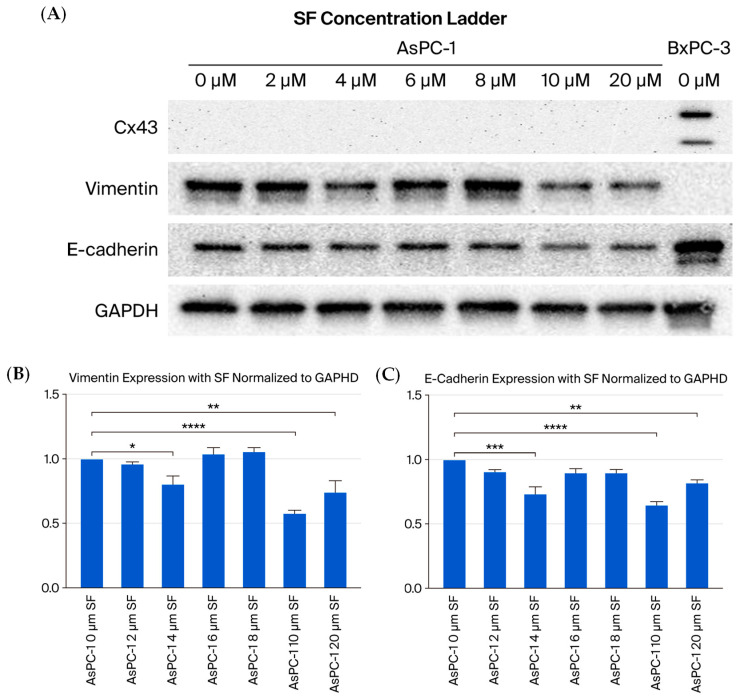
Dose-dependent effects of SF on Cx43 and EMT marker expression in AsPC-1 cells treated with 0–20 µM SF. (**A**) Representative Western blot images of vimentin, E-cadherin, Cx43, and GAPDH. Cx43 was undetectable across all SF concentrations in AsPC-1 cells; an untreated BxPC-3 sample is included as a positive control for Cx43 expression. (**B**) Relative vimentin expression normalized to GAPDH and expressed as fold change relative to untreated control (0 µM SF = 1.0). Expression decreased significantly at 4 µM (* *p* = 0.0293), 10 µM (**** *p* < 0.0001), and 20 µM (** *p* = 0.0041) SF. (**C**) Relative E-cadherin expression normalized to GAPDH. Expression decreased significantly at 4 µM (*** *p* = 0.0002), 10 µM (**** *p* < 0.0001), and 20 µM (** *p* = 0.0046) SF, with the most pronounced reduction at 10 µM, suggesting concentration-dependent modulation of E-cadherin by SF. Statistical significance was determined via one-way ANOVA with Dunnett’s multiple comparisons test. * *p* < 0.05, ** *p* < 0.01, *** *p* < 0.001, **** *p* < 0.0001.

To investigate potential mechanisms underlying SF-mediated enhancement of cytotoxicity, we examined whether SF could modulate Cx43 expression and EMT markers in AsPC-1 cells across a range of concentrations (2–20 µM). Western blot analysis revealed that Cx43 was undetectable across all SF concentrations tested in AsPC-1 cells, including at 10 µM (Figure 6A). In contrast, Cx43 was clearly expressed in the BxPC-3 control, consistent with its pre-metastatic phenotype. While the absence of Cx43 in AsPC-1 cells aligns with the loss of connexin expression commonly observed in metastatic cancers, we were unable to restore Cx43 expression under our experimental conditions. We next examined the effects of SF on vimentin and E-cadherin. Vimentin expression exhibited a non-linear, dose-dependent response, showing minimal change at 2 µM SF, returning to near baseline at moderate doses (6–8 µM), but decreasing significantly at 10 µM SF (0.58 ± 0.05, **** *p* < 0.0001), before partially recovering at 20 µM SF (0.74 ± 0.16, ** *p* = 0.0041) (Figure 6B, Table 3). E-cadherin showed a similar pattern, with significant reductions at 4 µM SF (0.73 ± 0.09, *** *p* = 0.0002) and the most pronounced reduction at 10 µM SF (0.65 ± 0.04, **** *p* < 0.0001), before partially recovering at 20 µM SF (0.82 ± 0.04, ** *p* = 0.0046) (Figure 6C, Table 3). The parallel suppression of both markers at 10 µM SF suggests a broader destabilization of EMT-related proteins rather than a specific reversal of the mesenchymal phenotype.

While 10 µM SF emerged as the most effective concentration for modulating EMT marker expression, the simultaneous suppression of both epithelial (E-cadherin) and mesenchymal (vimentin) markers, combined with the absence of Cx43 restoration, suggests that SF does not induce a clear reversal to a pre-metastatic phenotype in AsPC-1 cells. SF modulates these markers in a dose-dependent manner, though not through the anticipated mechanism of phenotype reversal, and the precise mechanisms underlying these effects remain to be elucidated.

#### 3.2.3. Sulforaphane Modulation of EMT Markers Varies in NTP + TPZ Combination Therapy

**Figure 7:**
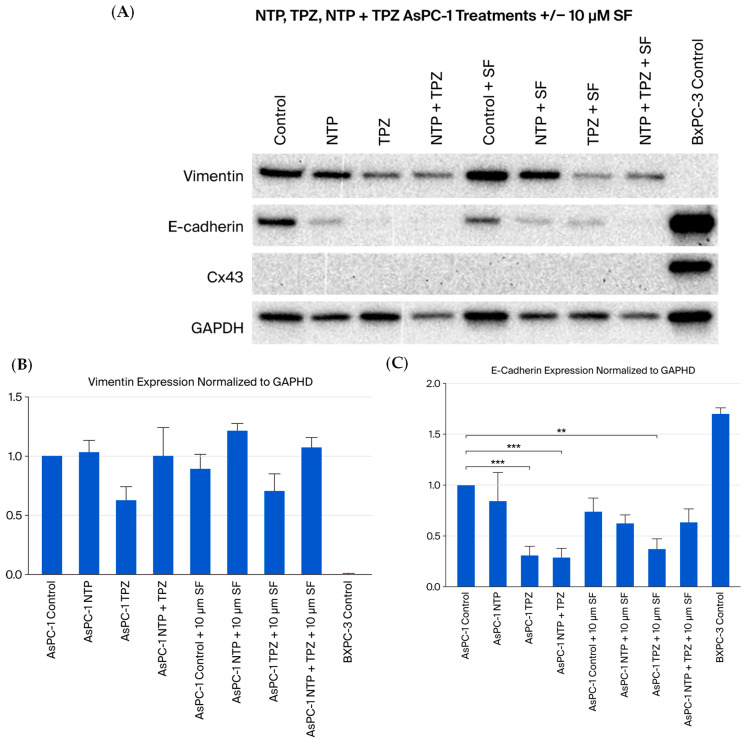
Western blot analysis and densitometric quantification of EMT marker expression in AsPC-1 cells treated with NTP, TPZ, or NTP + TPZ (N/T) in the absence or presence of 10 µM SF. (**A**) Representative Western blot images of vimentin, E-cadherin, Cx43, and GAPDH. An untreated BxPC-3 sample is included as a positive control for Cx43 expression. (**B**) Relative vimentin expression normalized to GAPDH and expressed as fold change relative to untreated control (set to 1.0). Vimentin expression remained statistically unchanged across all conditions, suggesting it was not significantly modulated by SF in the context of NTP and TPZ combination treatment. (**C**) Relative E-cadherin expression normalized to GAPDH. E-cadherin was significantly reduced following TPZ (*** *p* = 0.0007) and NTP + TPZ (*** *p* = 0.0005), with suppression maintained in TPZ + SF (** *p* = 0.002), indicating that SF preserved rather than mitigated TPZ-driven suppression of this epithelial marker. Adding SF to NTP + TPZ resulted in no significant difference from control. Statistical significance was determined via one-way ANOVA with Dunnett’s multiple comparisons test. ** *p* < 0.01, *** *p* < 0.001.

To further investigate the effects of SF on EMT markers in the context of NTP + TPZ treatment, we examined vimentin and E-cadherin expression in AsPC-1 cells treated with NTP, TPZ, or NTP + TPZ in the absence or presence of 10 µM SF (Figure 7A, Table 4). Vimentin expression showed variable responses across treatment conditions (Figure 7B, Table 4). The high variability and lack of statistical significance in vimentin expression changes suggest that SF’s effects on vimentin are context-dependent and differ when combined with NTP + TPZ compared to SF treatment alone. Unlike vimentin, E-cadherin expression changes reached statistical significance in several treatment conditions (Figure 7C, Table 4). TPZ and NTP + TPZ treatments significantly reduced E-cadherin expression to 0.31 ± 0.08 (*** *p* = 0.0007) and 0.29 ± 0.08 (*** *p* = 0.0005), respectively. The addition of SF to TPZ maintained this suppression (0.37 ± 0.10, ** *p* = 0.002), while adding SF to NTP + TPZ resulted in no significant difference from control, suggesting that SF’s effects on E-cadherin depend on the treatment context.

## 4. Discussion

### 4.1. Overview of Key Findings

This study demonstrates that NTP and TPZ combine to produce cytotoxic effects exceeding either single agent alone in both pre-metastatic and metastatic pancreatic adenocarcinoma cells, with near-complete elimination of metastatic AsPC-1 cells. Bliss independence analysis confirmed additive to synergistic interactions depending on cell line and treatment combination. The addition of SF significantly enhanced this cytotoxicity across all treatment conditions. Notably, the cytotoxic enhancement by SF occurred without restoration of Cx43 expression or coordinated reversal of EMT markers. These findings support NTP + TPZ as a promising therapeutic approach for pancreatic cancer and identify SF as a potent adjunct that enhances efficacy through alternative molecular pathways that remain to be fully characterized.

### 4.2. NTP and TPZ Combination Mechanisms in Pancreatic Cancer

The additive to synergistic cytotoxicity observed between NTP and TPZ is consistent with their complementary mechanisms of action. NTP generates reactive oxygen and nitrogen species (RONS) that induce oxidative damage and trigger apoptotic pathways in cancer cells [19], creating a cellular environment that enhances the efficacy of TPZ, a bioreductive prodrug activated under hypoxic conditions [20]. This approach is particularly relevant for pancreatic adenocarcinoma, where chronic hypoxia is a defining characteristic of the tumor microenvironment and contributes to treatment resistance [21,22], as hypoxic conditions simultaneously enhance TPZ activation while limiting the effectiveness of conventional oxygen-dependent therapies. Similar oxidative stress and hypoxia-targeted combination approaches have demonstrated enhanced efficacy across multiple solid tumor types including glioblastoma and small cell lung cancer [23].

Our Bliss independence analysis confirmed approximately additive to moderately synergistic interactions depending on cell line (BxPC-3: Δ + 1.4%; AsPC-1: Δ + 12.9%), with the more pronounced effect in metastatic AsPC-1 cells consistent with their increased hypoxic adaptation favoring TPZ activation. While gap junction-mediated intercellular communication has been proposed to amplify NTP cytotoxicity through RONS transmission between cells [19,24], the strong cytotoxic effects achieved suggest that NTP and TPZ combination therapy remains highly effective through direct cytotoxic mechanisms even without gap junction enhancement.

### 4.3. Sulforaphane Enhancement Through Alternative Molecular Pathways

The addition of SF to NTP + TPZ treatment produced substantial enhancements in cytotoxicity across both cell lines, with Bliss independence analysis revealing synergistic effects in BxPC-3 cells (Δ + 27.8% for NTP + TPZ+SF) and additive to synergistic effects in AsPC-1 cells. These pronounced effects occurred despite the absence of Cx43 upregulation or coordinated EMT reversal, suggesting that SF enhances treatment efficacy through mechanisms distinct from gap junction modulation [25].

SF possesses multiple anticancer properties that may contribute to this enhancement, including activation of the Nrf2 pathway and induction of phase II detoxification enzymes [26,27], which may paradoxically enhance TPZ sensitivity by modulating cellular redox balance in ways that favor its reductive activation under hypoxic conditions. Beyond redox modulation, SF has been shown to suppress NF-κB signaling [28], sensitize cancer cells to apoptosis through caspase-3, -8, and -9 activation [29], and inhibit HIF-1α, thereby potentially impairing hypoxic survival mechanisms that contribute to treatment resistance in PDAC [30]. The convergence of these complementary actions may contribute to the substantial cytotoxicity observed, particularly in the metastatic AsPC-1 cell line, and the cytotoxic enhancement without Cx43 upregulation suggests SF acts through alternative mechanisms including its documented effects on ROS and cellular redox balance [31].

### 4.4. Cx43 and EMT: Discrepancies and Mechanistic Complexity

#### 4.4.1. Absence of Cx43 Upregulation: Implications for EMT Regulation

Cx43 was undetectable across all SF concentrations tested (2–20 µM) in AsPC-1 cells, despite prior work by Forster et al. demonstrating SF-induced Cx43 expression in this cell line through post-translational phosphorylation of GSK3 and JNK [12]. AsPC-1 cells are known to lack endogenous expression of multiple connexins including Cx32, Cx40, and Cx43 [12], and promoter hypermethylation represents a potential barrier that may explain this discrepancy, as hypermethylation of the Cx43 promoter has been shown to silence its expression in gliomas and lung cancer [32,33]. Specifically, inhibition of GSK3 and JNK has been found to prevent SF-induced Cx43 expression, while SF-induced phosphorylation changes have been shown to increase Cx43 expression under other conditions [12], suggesting that phosphorylation mechanisms under our experimental conditions may have been insufficient to overcome this epigenetic barrier. Variations in cell passage number or culture conditions may have further contributed to the divergent outcome.

The absence of Cx43 suggests that SF did not reverse the EMT phenotype under our experimental conditions, which is consistent with evidence that Cx43 deficiency can initiate EMT and induce drug insensitivity. For example, Cx43 knockdown induced EMT markers and cisplatin resistance in lung adenocarcinoma cells [34], and Cx43 deficiency conferred tamoxifen resistance through EMT modulation in breast cancer cells where Cx43 overexpression restored an epithelial phenotype [35]. These findings suggest that loss of Cx43 may maintain the mesenchymal state, potentially explaining why vimentin suppression was not observed as hypothesized.

#### 4.4.2. E-Cadherin and N-Cadherin Modulation: Alternative Roles and Absence of Cadherin Switch

Our analysis of EMT markers revealed context-dependent effects that did not support a coordinated phenotype reversal. E-cadherin expression appeared reduced in both cell lines following treatment, with AsPC-1 cells showing notably lower baseline expression consistent with their metastatic phenotype and known reduced cell–cell adhesion [36]. The classic cadherin switch involves loss of E-cadherin accompanied by N-cadherin upregulation to facilitate cell migration [37]. However, N-cadherin upregulation was not observed under any treatment conditions, suggesting that our treatments did not induce the full EMT program.

The TPZ-mediated reduction in E-cadherin may instead reflect disruption of alternative E-cadherin-mediated survival mechanisms, as E-cadherin has been shown to paradoxically promote metastasis by limiting ROS-mediated apoptosis in invasive ductal carcinoma [18] and to upregulate the de novo serine synthesis pathway to support oxidative stress resistance [38], suggesting its reduction may sensitize cells to cytotoxic death rather than promote invasion. The absence of the cadherin switch, which may be related to the lack of Cx43 expression given its role in stabilizing epithelial markers and suppressing mesenchymal transitions [37], represents a therapeutically favorable outcome as even partial EMT can contribute to increased invasiveness [39], and our findings suggest NTP + TPZ reduces cell viability without evidence of enhancing invasive characteristics in vitro.

#### 4.4.3. Context-Dependent Vimentin Responses and Sulforaphane’s Dual Role in Redox Regulation

While SF alone significantly reduced both vimentin and E-cadherin expression, the addition of NTP and TPZ altered these responses, with SF either causing no change or increasing vimentin expression across all combination treatment groups. These variable effects differ from studies demonstrating SF-mediated EMT inhibition through Nrf2 activation and TGF-β suppression in other cancer models [40,41], highlighting the influence of treatment context on SF’s effects.

A likely explanation involves SF’s dual role in redox regulation: while it activates Nrf2 to induce antioxidant defenses [26,27], SF has also been shown to increase intracellular ROS in cancer cells [42], and in the context of our experiments where NTP and TPZ already impose substantial oxidative stress [7,11], SF may further elevate ROS levels in ways that modulate cytoskeletal proteins such as vimentin [31,43]. Importantly, these variable vimentin responses were not accompanied by N-cadherin upregulation or other evidence of coordinated EMT progression, suggesting they reflect stress-associated cytoskeletal responses rather than a transition toward increased invasiveness.

### 4.5. Study Limitations and Future Directions

This study was conducted using two-dimensional cell culture, which does not fully capture the structural and metabolic complexity of pancreatic tumors, including spatial hypoxia gradients and three-dimensional cell–cell interactions that influence therapeutic response in vivo. Extension into three-dimensional culture systems such as spheroids or patient-derived organoids represents a planned next step, consistent with our laboratory’s established trajectory of progressing from two-dimensional models into more complex systems. Future studies would also benefit from broader molecular profiling of stress-responsive signaling pathways and EMT regulatory transcription factors such as Snail, Slug, and Twist, which could help identify predictive biomarkers informing responsiveness to this treatment strategy. Given SF’s established human safety profile and TPZ’s prior clinical trial history, continued investigation of this triple combination therapy in more advanced model systems represents a feasible and clinically relevant direction.

## 5. Conclusions

Due to the highly aggressive nature of pancreatic adenocarcinoma and limited treatment options, the novel combination therapy of NTP and TPZ has demonstrated significant cytotoxic success in vitro, which warrants further investigation. The addition of sulforaphane substantially enhanced this cytotoxic effect in both pre-metastatic and metastatic pancreatic cancer cells without inducing invasive characteristics in vitro. This early in vitro study identifies a potential treatment modality for pancreatic adenocarcinoma that can be tested in animal models, which may ultimately inform the development of novel therapeutic strategies for this devastating disease.

## Figures and Tables

**Table 1:** Cell viability of BxPC-3 and AsPC-1 cells following treatment with NTP, TPZ, or NTP + TPZ, expressed as mean percent viability relative to untreated control. Statistical significance was assessed via one-way ANOVA with Dunnett’s multiple comparisons test. ** *p* < 0.01, **** *p* < 0.0001. Significant decreases in viability were observed across all treatment conditions in both cell lines, with the strongest effects observed for the NTP + TPZ combination.

Cell Line	Treatment	Percent Viability (Mean)	Adjusted *p*-Value	Significance
BxPC-3	Control	100.0	-	-
	NTP	30.3	<0.0001	****
	TPZ	60.0	0.0087	**
	NTP + TPZ	12.7	<0.0001	****
AsPC–1	Control	100.0	-	-
	NTP	39.5	<0.0001	****
	TPZ	5.5	<0.0001	****
	NTP + TPZ	0.48	<0.0001	****

**Table 2:** Bliss independence analysis of SF combinations in BxPC-3 and AsPC-1 cells. Predicted fractional inhibition was calculated using the Bliss independence model as E(A + B) = E(A) + E(B) − E(A) × E(B), where E(A) and E(B) represent the fractional inhibition of each single agent. Positive Δ values indicate synergy, values near zero indicate additivity, and negative Δ values indicate antagonism. In BxPC-3 cells, SF showed primarily additive effects with NTP and TPZ alone but strong synergy with the NTP + TPZ combination (Δ + 27.8%). In AsPC-1 cells, SF showed moderate synergy with NTP (Δ + 9.2%), antagonism with TPZ (Δ − 10.3%), and additive effects with NTP + TPZ (Δ + 0.7%), with overall enhancement of cytotoxicity observed across all conditions.

Treatment Combination	Observed Inhibition (%)	Predicted Inhibition (%)	Δ (Observed − Predicted)
	BxPC-3		
N/T inhibition	68.42654	67.00939	1.417144
NTP + SF inhibition	87.53039	82.28642	5.243972
TPZ + SF inhibition	69.3991	68.44724	0.951857
N/T + SF inhibition	86.6273	58.83987	27.78743
	AsPC-1		
N/T inhibition	70.09901	57.19962	12.89939
NTP + SF inhibition	95.0495	85.80544	9.244061
TPZ + SF inhibition	69.43894	79.70983	−10.2709
N/T + SF inhibition	92.93729	92.24351	0.693788

**Table 3:** Dose-dependent effects of SF on EMT marker expression in AsPC–1 cells, normalized to GAPDH. Statistical significance was determined via one-way ANOVA followed by Dunnett’s multiple comparisons test (GraphPad Prism, version 10). *p*-values are reported after adjustment for multiple comparisons. * *p* < 0.05, ** *p* < 0.01, *** *p* < 0.001, **** *p* < 0.0001. Treatment with 10 µM SF produced the strongest and most significant reductions in both vimentin and E-cadherin expression. Additional decreases were observed at 4 µM SF for both markers and at 20 µM SF for E-cadherin, though these effects were less pronounced. Other concentrations showed no significant changes.

Treatment	Vimentin (Ratio ± SEM)	*p*-Value	E-Cadherin (Ratio ± SD)	*p*-Value
AsPC–1 0 µM SF	1.00 ± 0.00	–	1.00 ± 0.00	–
AsPC–1 2 µM SF	0.96 ± 0.03	0.9626	0.91 ± 0.03	0.1854
AsPC–1 4 µM SF	0.81 ± 0.11	* 0.0293	0.73 ± 0.09	*** 0.0002
AsPC–1 6 µM SF	1.04 ± 0.10	0.9759	0.90 ± 0.06	0.1248
AsPC–1 8 µM SF	1.05 ± 0.07	0.8571	0.89 ± 0.05	0.1102
AsPC–1 10 µM SF	0.58 ± 0.05	**** <0.0001	0.65 ± 0.04	**** <0.0001
AsPC–1 20 µM SF	0.74 ± 0.16	** 0.0041	0.82 ± 0.04	** 0.0046

**Table 4:** Expression changes in EMT markers in AsPC–1 cells normalized to GAPDH. Statistical significance was determined via one-way ANOVA followed by Dunnett’s multiple comparisons test (GraphPad Prism, version 10). *p*-values are reported after adjustment for multiple comparisons. *p* < 0.01 = **, *p* < 0.001 = ***. Treatment with NTP, TPZ, and NTP + TPZ did not result in statistically significant reductions in vimentin expression, although TPZ alone exhibited a downward trend (*p* = 0.111). Significant reductions in E-cadherin expression were observed with TPZ alone (*p* = 0.0007) and NTP + TPZ (*p* = 0.0005).

Treatment	Vimentin (Ratio ± SEM)	*p*-Value	E-Cadherin (Ratio ± SEM)	*p*-Value
Control	1.00 ± 0.00	-	1.00 ± 0.00	-
NTP	1.03 ± 0.10	>0.9999	0.84 ± 0.28	0.866
TPZ	0.62 ± 0.11	0.111	0.31 ± 0.08	*** 0.0007
NTP + TPZ	1.00 ± 0.23	>0.9999	0.29 ± 0.08	*** 0.0005
Control + 10 µM SF	0.90 ± 0.12	0.984	0.73 ± 0.14	0.404
NTP + 10 µM SF	1.21 ± 0.07	0.647	0.62 ± 0.08	0.113
TPZ + 10 µM SF	0.71 ± 0.14	0.310	0.37 ± 0.10	** 0.002
NTP + TPZ + 10 µM SF	1.07 ± 0.08	0.999	0.63 ± 0.13	0.119

## Data Availability

The original contributions presented in this study are included in the article. Further inquiries can be directed to the corresponding author.
